# Supplementary material for: Dendritic Cells of Leukemic Origin (DCleu) Modulate the Expression of Inhibitory Checkpoint Molecules and Their Ligands on T Cells and Blasts in AML Relapse After Allogeneic Stem Cell Transplantation
Source: Cancers (Basel). 2025 Sep 9;17(18):2948. doi: 10.3390/cancers17182948 (PMC12468131; doi:10.3390/cancers17182948)
Supplement: Supplementary file 1 [file cancers-17-02948-s001.zip › cancers-3760210-supplementary.pdf]

Supplementary Materials for

Dendritic Cells of Leukemic Origin (DC_{leu}) Modulate the Expressions of Inhibitory Checkpoint Molecules and Their Ligands on T Cells and Blasts in AML Relapse After Allogeneic Stem Cell Transplantation

Xiaojia Feng [1,2,*,†], Giuliano Filippini Velázquez [2,3,†], Sophia Bohlscheid [1,2,†], Marianne Unterfrauner [1,2], Philipp Anand [1,2], Hazal Aslan Rejeski [1,2], Anne Hartz [1,2], Tobias Baudrexler [1,2], Christoph Schmid [2,3,‡] and Helga Maria Schmetzer [1,2,*,‡]

1. Department of Medicine III, University Hospital of Munich, 81377 Munich, Germany

2. Bavarian Center for Cancer Research (BZKF), 91054 Erlangen, Germany

3. Department of Hematology and Oncology, Augsburg University Hospital and Medical Faculty, 86156 Augsburg, Germany

† These authors contributed equally to this work.
‡ These authors contributed equally to this work.

Table S1 Patients' characteristics

Patient No.	Age/ Sex	Diagnose (WHO)	Last Nr of allo-HCT	Donor type	ELN 2022-Risk-Classification (at diagnosis)	Last Nr of allo-HCT	Cytogenetics	Molecular genetics (at relapse)	Blast phenotype (CD)	BM/ WB Blasts [%]	Salvage treatment	Response to initial salvage treatment	Experiments Conducted with WB
1628	23/F	AML	allo-HCT2	Haplo	Adverse	allo-HCT2	Complex aberrant karyotype	RUNX1, WT1, FLT3-ITD, KMT2A-PTD	**117**,34,33,56	46/4.5	Aza. /Deci. + Ven. (2x)	No	DC, MLC, Deg (UC,C), InCyt (UC,C), CTX
1632	57/F	AML	allo-HCT2	MUD	Adverse	allo-HCT2	Complex aberrant karyotype, RUNX1 rearrangement, MECOM rearrangement	na.	**34**,13,33,117	57/61	Deci. +Ven. (1x), low-dose AraC	No	DC, MLC, Deg (UC,C), InCyt (UC,C), CTX
1640	73/F	AML	allo-HCT1	MUD	Favorable	allo-HCT1	9q-Deletion	DNMT3A, NMP1, WT1, IDH2	**117**,33,7,34	Ps./1	Aza. + Ven. (6x)	Yes	DC, MLC, Deg (UC,C), InCyt (UC,C), CTX
1641	65/F	AML	allo-HCT1	MUD	Intermediate	allo-HCT1	dup13(q12q33), DLEU-Signal	FLT3-ITD, RUNX1, WT1	**117**,34,33,65	45/5	Aza/Deci. + Ven. (2x)	No	DC, MLC, Deg (UC,C), InCyt (UC,C), CTX
1650	64/F	AML	allo-HCT1	MUD	Adverse	allo-HCT1	na.	RUNX1, TP53, STAG2, RUNX1	**117**,34,13	21/0.65	Aza. + Ven. (6x)	Yes	DC, MLC, Deg (UC,C), InCyt (UC,C), CTX
1654	71/M	AML	allo-HCT1	MUD	Favorable	allo-HCT1	der16, t(16;17)	NPM1	**117**,34,13	<5/2.9	Aza. + Ven. (5x)	Yes	DC, MLC, Deg (UC,C), InCyt (UC,C), CTX
1655	65/M	AML	allo-HCT1	MUD	Adverse	allo-HCT1	46, XY	IDH2, RUNX1, ASXL1	**117**,34,33,56	80/5.4	Deci. + Ven. (2x)	No	DC, MLC, Deg (UC,C), InCyt (UC,C), CTX
1656	43/F	AML	allo-HCT1	Haplo	Adverse	allo-HCT1	Complex aberrant karyotype, MECOM rearrangement	PTPN11	**34**,117,13,33	50/9	Deci.+ Vene. (1x)	No	DC, MLC, Deg (UC,C), InCyt (UC,C), CTX

1658	60/M	AML	allo-HCT1	MSD	Intermediate	allo-HCT1	Trisomy 6,6q21, 6q23 signal	KRAS, FLT3-TKD, CEBPA	**13**,34,117,33	64/4.6	Deci. + Ven. (1x)	No	DC, MLC, Deg (UC,C), InCyt (UC,C), CTX
1660	71/M	MDS	allo-HCT1	MUD	na.	allo-HCT1	5q-del., Monosomy7	NF1	**117**,34,33,13	<5/5	Aza. +Ven. (7x)	Yes	DC, MLC, Deg (UC,C), InCyt (UC,C), CTX
1663	46/F	AML	allo-HCT2	MUD	Intermediate	allo-HCT2	Trisomy6, 6q21-, 6q23 signal	FLT3-ITD, RUNX1, ASXL1, WT1	**34**,117,33,13	80/10.2	Cyt. + Deci. + Ven. (2x)	Yes	DC, MLC, Deg (UC,C), InCyt (UC,C), CTX
1664	65/M	AML	allo-HCT2	MUD	Adverse	allo-HCT2	7q31-del., ETV6 Del	BCR/ABL1, RUNX1, KRAS, FLT3-ITD	**117**,34,13,33	Ps./3	Aza. (4x)	No	DC, MLC, Deg (UC,C), InCyt (UC,C), CTX
1665	59/M	AML	allo-HCT1	MUD	Adverse	allo-HCT1	KMT2A-signal, Trisomy11	TET2, BCOR, STAG2, ZRSR2	**117**,33,13,34	<5%/2	Aza. + Ven. (5x)	Yes	DC, MLC, Deg (UC,C), InCyt (UC,C), CTX
1673	53/M	AML	allo-HCT1	MUD	Adverse	allo-HCT1	46, XY	RUNX1, BCOR, DNMT3A, IDH2	**34**,117,13,33	80/30	Cyt., Mito., Ven. (1x)	Yes	DC, MLC, Deg (UC,C), InCyt (UC,C), CTX
1674	52/F	AML	allo-HCT1	Haplo	Adverse	allo-HCT1	Complex aberrant karyotype, KMT2A-signal, 5p15-del., 5q31-del., 7q31-del., ETV6-del.	TP53	**34**,117,13,33	Ps./7	Deci. (3x)	No	DC, MLC, Deg (UC,C), InCyt (UC,C), CTX

Legend: F: female; M: male; AML: acute myeloid leukemia; MDS: Myelodysplastic syndrome; WHO: World Health Organization; ELN: European Leukemia Network; allo-HCT: Allogeneic hematopoietic stem cell transplantation; Haplo: Haploidentical Donors; MUD: Matched Unrelated Donors; MSD: Matched Sibling Donors; na.: not applicable; del.: deletion; t: translocation; der: derivative; ampl: amplification; dup: duplication; CD: Cluster of differentiation; bold: antibody used for expression analyses; WB: Whole blood; BM: bone marrow; nd.:no data; Ps.:Punctio sicca; Aza.: Azacitidin; Vene.: Venetoclax; Deci.: Decitabin; Cyt.: Cytarabine; Mito.: Mitoxantrone; DC: dendritic cell culture measurements; MLC: mixed lymphocyte culture measurement; Deg: degranulation assay; InCyt: intracellular cytokine assay; CTX: cytotoxicity measurements; UC: measurements in uncultured samples; C: measurements after mixed lymphocyte culture;

Table S2: Cells and cell subsets as evaluated by flow cytometry.

Cell Type	Name of Subgroups	Abbreviation of Subgroups	Surface Marker	Referred to	Abbreviation
**Blast cells**	Blasts	BLA	BLA+ e.g. CD34+, CD117+	WB (whole blood)	BLA/WB
	Proliferating blasts	$BLA_{prol-CD71}$	BLA+DC-CD71+	BLA	$BLA_{prol-CD71}$/BLA
	Proliferating blasts	$BLA_{prol-IPO38}$	BLA+DC-IPO38+	BLA	$BLA_{prol-IPO38}$/BLA
	Immune Checkpoint molecules (ICM)	BLA_{CTLA4}	BLA+CD152+	BLA	BLA_{CTLA4}/BLA
		BLA_{PD1}	BLA+CD279+	BLA	BLA_{PD1}/BLA
		BLA_{PDL1}	BLA+CD274+	BLA	BLA_{PDL1}/BLA
		BLA_{PDL2}	BLA+CD273+	BLA	BLA_{PDL2}/BLA
		BLA_{TIM3}	BLA+TIM3+	BLA	BLA_{TIM3}/BLA
**Monocytoid cells**	Proliferating CD14+ monocytes	$Mon_{prol-CD71}$	CD14+DC-CD71+	CD14+	$Mon_{prol-CD71}$/Mon
	Proliferating CD14+ monocytes	$Mon_{prol-IPO38}$	CD14+DC-IPO38+	CD14+	$Mon_{prol-IPO38}$/Mon
**Dendritic cells**	Dendritic cells	DC	DC+ e.g. CD80+, CD206+	WB	DC/WB
	Leukaemia-derived DC	DC_{leu}	DC+BLA+	WB or DC or BLA	DC_{leu}/WB<br>DC_{leu}/BLA
	Mature migratory DC	DC_{mat}	DC+CD197+	WB or DC	DC_{mat}/WB<br>DC_{mat}/DC
	Mature migratory DC_{leu}	$DC_{mat+leu}$	DC+BLA+CD197+	WB or DC or DC_{leu} oder DC_{mat} or BLA	$DC_{leu-mat}$/WB<br>$DC_{leu-mat}$/DC<br>$DC_{leu-mat}$/BLA
	Immune Checkpoint molecules (ICM)	DC_{CTLA4+}	DC+CD152+	DC	DC_{CTLA4+}/DC

		DC_{PD1+}	DC+CD279+	DC	DC_{PD1+}/DC
		DC_{PDL1+}	DC+CD274+	DC	DC_{PDL1+}/DC
		DC_{PDL2+}	DC+CD273+	DC	DC_{PDL2+}/DC
		DC_{TIM3+}	DC+TIM3+	DC	DC_{TIM3+}/DC
		DC_{KLRG1+}	DC+KLRG1+	DC	DC_{KLRG1+}/DC
		DC_{LAG3+}	DC+LAG3+	DC	DC_{LAG3+}/DC
		DC_{2B4+}	DC+2B4+	DC	DC_{2B4+}/DC
		DC_{TIGIT+}	DC+TIGIT+	DC	DC_{TIGIT+}/DC
**T lymphocytes**	CD3+ pan T cells	T_{CD3+}	CD3+	lymphocytes	T_{CD3+}/WB
	CD3+CD4+T cells	$T_{CD3+CD4+}$	CD3+CD4+	lymphocytes	$T_{CD3+CD4+}/WB$
	CD3+CD4- T cells	$T_{CD3+CD4-}$	CD3+CD4-	lymphocytes	$T_{CD3+CD4-}/WB$
	Non-naive T cells	$T_{non-naive}$	CD3+CD45RO+	CD3+	$T_{non-naive}/T_{CD3+}$
	Central memory T cells	T_{cm}	CD3+CD45RO+CD197+	CD3+	T_{cm}/T_{CD3+}
	Effector memory T cells	$T_{em/eff}$	CD3+CD45RO+CD197-	CD3+	$T_{em/eff}/T_{CD3+}$
	Proliferating T cells - late	$T_{prol-late}$	CD3+CD71+	CD3+	$T_{prol-late}/ T_{CD3+}$
	CTLA4+	$T_{CD3+CTLA4+}$	CD3+CD152+	CD3+	$T_{CD3+CTLA4+}/T_{CD3+}$
		$T_{CD3+CD4+CTLA4+}$	CD3+CD4+CD152+	CD3+CD4+	$T_{CD3+CD4+CTLA4+}/T_{CD3+CD4+}$
		$T_{CD3+CD4-CTLA4+}$	CD3+CD4-CD152+	CD3+CD4-	$T_{CD3+CD4-CTLA4+}/T_{CD3+CD4-}$
	PD1	$T_{CD3+PD1+}$	CD3+CD279+	CD3+	$T_{CD3+PD1+}/T_{CD3+}$
		$T_{CD3+CD4+PD1+}$	CD3+CD4+CD279+	CD3+CD4+	$T_{CD3+CD4+PD1+}/T_{CD3+CD4+}$
		$T_{CD3+CD4-PD1+}$	CD3+CD4-CD279+	CD3+CD4-	$T_{CD3+CD4-+PD1+}/T_{CD3+CD4-}$
	PDL1	$T_{CD3+PDL1+}$	CD3+CD274+	CD3+	$T_{CD3+PDL1+}/T_{CD3+}$
		$T_{CD3+CD4+PDL1+}$	CD3+CD4+CD274+	CD3+CD4+	$T_{CD3+CD4-PDL1+}/T_{CD3+CD4-}$

		$T_{CD3+CD4-PDL1+}$	CD3+CD4-CD274+	CD3+CD4-	$T_{CD3+CD4-PDL1+}$ / $T_{CD3+CD4-}$
	PDL2	$T_{CD3+PDL2+}$	CD3+CD273+	CD3+	$T_{CD3+PDL2+}/T_{CD3+}$
	TIGIT	$T_{CD3+TIGIT+}$	CD3+TIGIT+	CD3+	$T_{CD3+TIGIT+}/T_{CD3+}$
		$T_{CD3+CD4+TIGIT+}$	CD3+CD4+TIGIT+	CD3+CD4+	$T_{CD3+CD4+TIGIT+}/T_{CD3+CD4+}$
		$T_{CD3+CD4-TIGIT+}$	CD3+CD4-TIGIT+	CD3+CD4-	$T_{CD3+CD4-TIGIT+}/T_{CD3+CD4-}$
	2B4	$T_{CD3+2B4+}$	CD3+2B4+	CD3+	$T_{CD3+2B4+}/T_{CD3+}$
		$T_{CD3+CD4+2B4+}$	CD3+CD4+2B4+	CD3+CD4+	$T_{CD3+CD4+2B4+}/T_{CD3+CD4+}$
		$T_{CD3+CD4-2B4+}$	CD3+CD4-2B4+	CD3+CD4-	$T_{CD3+CD4-2B4+}/T_{CD3+CD4-}$
	KLRG1	$T_{CD3+KLRG1+}$	CD3+KLRG1+	CD3+	$T_{CD3+KLRG1+}/T_{CD3+}$
		$T_{CD3+CD4+KLRG1+}$	CD3+CD4+KLRG1+	CD3+CD4+	$T_{CD3+CD4+KLRG1+}/T_{CD3+CD4+}$
		$T_{CD3+CD4-KLRG1+}$	CD3+CD4-KLGR1+	CD3+CD4-	$T_{CD3+CD4-KLRG1+}/T_{CD3+CD4-}$
	TIM3	$T_{CD3+TIM3+}$	CD3+TIM3+	CD3+	$T_{CD3+TIM3+}/T_{CD3+}$
		$T_{CD3+CD4+TIM3+}$	CD3+CD4+TIM3+	CD3+CD4+	$T_{CD3+CD4+TIM3+}/T_{CD3+CD4+}$
		$T_{CD3+CD4-TIM3+}$	CD3+CD4-TIM3+	CD3+CD4-	$T_{CD3+CD4-TIM3+}/T_{CD3+CD4-}$
	LAG3	$T_{CD3+TIM3+}$	CD3+LAG3+	CD3+	$T_{CD3+TIM3+}/T_{CD3+}$
		$T_{CD3+CD4+LAG3+}$	CD3+CD4+LAG3+	CD3+CD4+	$T_{CD3+CD4+LAG3+}/T_{CD3+CD4+}$
		$T_{CD3+CD4-LAG3+}$	CD3+CD4-LAG3+	CD3+CD4-	$T_{CD3+CD4-LAG3+}/T_{CD3+CD4-}$
**B lymphocyte cells**	CD19+ B cells	degB/B	CD19+CD107a+	Bcell	degB/B
**T lymphocyte cells**	CD3+ pan T cells	$degT_{CD3+}/T_{CD3+}$	CD3+CD107a+	T_{3+}	$degT_{CD3+}/T_{CD3+}$
		$IFN\gamma{+}T_{CD3+}/T_{CD3+}$	CD3+IFNγ+	T_{3+}	$IFN\gamma{+}T_{CD3+}/T_{CD3+}$
	Non-naive T cells	$degT_{non\text{-}naive}$	CD3+CD45RO+CD107a+	$T_{non\text{-}naïve}$	$degT_{non\text{-}naive}/T_{non\text{-}naive}$
		$IFN\gamma{+}T_{non\text{-}naive}$	CD3+CD45RO+IFNγ+	$T_{non\text{-}naive}$	$IFN\gamma{+}T_{non\text{-}naïve}/T_{non\text{-}naive}$
	Central memory T cells	$degT_{cm}$	CD3+CD45RO+CD197+CD107a+	T_{cm}	$degT_{cm}/T_{cm}$

			IFNγ+T_{cm}	CD3+CD45RO+CD197+IFNγ+	T_{cm}	IFNγ+T_{cm}/T_{cm}
		Effector memory T cells	deg$T_{em/eff}$	CD3+CD45RO+CD197-CD107a+	$T_{em/eff}$	deg$T_{em/eff}$/$T_{em/eff}$
			IFNγ+$T_{em/eff}$	CD3+CD45Ro+CD197+IFNγ+	$T_{em/eff}$	IFNγ$T_{em/eff}$/$T_{em/eff}$
		Regulatory T cells	deg$T_{CD4+reg}$	CD4+CD25+CD127^{low}	CD4^{+}T_{reg}	deg$T_{CD4+reg+}$/$T_{CD4+reg+}$
		Integrinβ7^{+}coexpressing T cells	IFNγ+$T_{CD3+ß+}$	CD3+Integrinß7+IFNγ+	CD3+ß7+	IFNγ+$T_{CD3+ß7+}$/$T_{CD3+ß7+}$
	**Cytokine-induced killer cells**	CD3+CD56+ CIK cells	degCIK	CD3+CD56+CD107a+	CIKcell	degCIK/CIK
			IFNγ+CIK	CD3+CD56+IFNγ+	CIKcell	IFNγ+CIK/CIK
**Innate immune system**	**Natural killer cells**	CD3-CD56+ NK cells	degNK	CD3-CD56+CD107a+	NKcell	degNK/NK
			IFNγ+NK	CD3-CD56+IFNγ+	NKcell	IFNγ+NK/NK

Supplementary figures

Figure S1A. CTLA4 and PD1 expressing uncultured AML patients´ blasts

P1655

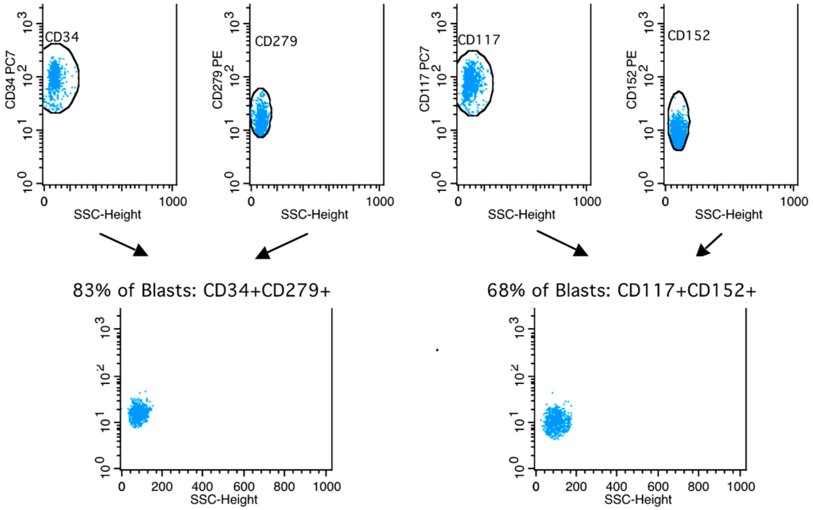

Figure S1B. ICM expressing uncultured/cultured AML patients´ T cells, DCs

P1674

TIGIT-expressing uncultured T cells **TIGIT-expressing T cells after MLC**

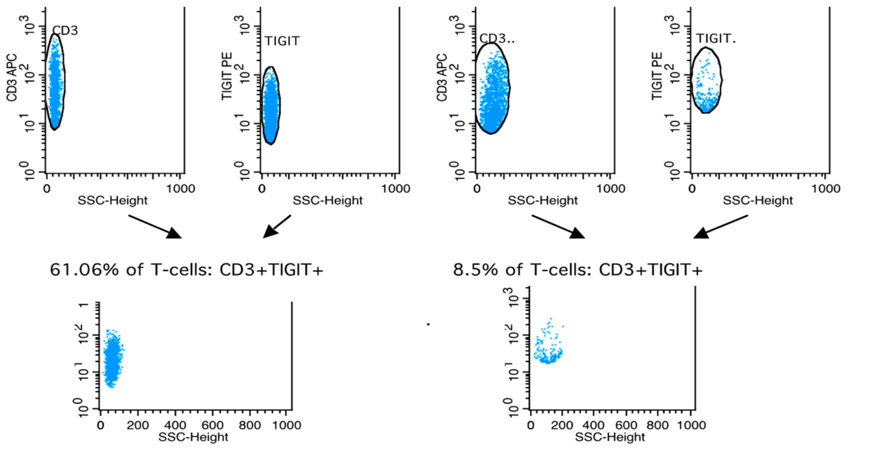

TIM3-expressing uncultured T cells **TIM3-expressing T cells after MLC**

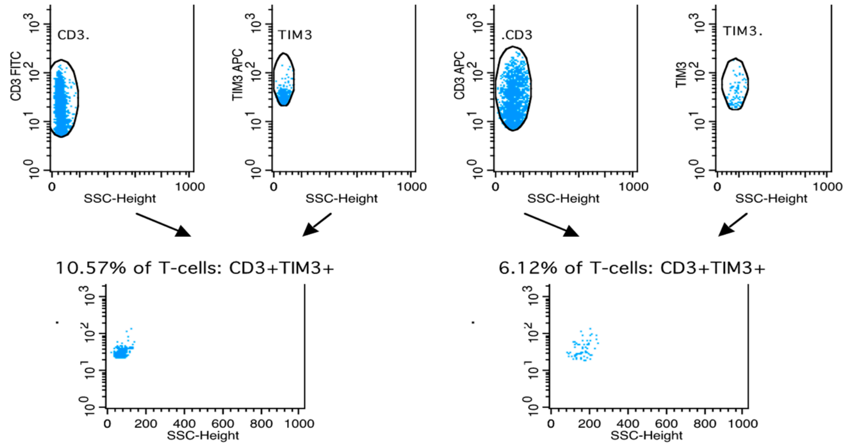

P1655

2B4 and CD152-expressing DCs after Kit-M culture

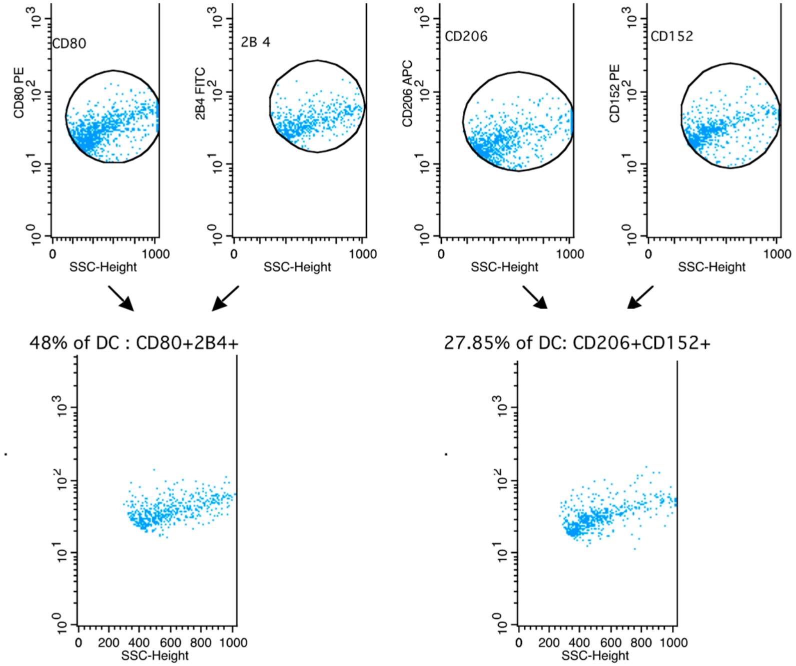

Figure S2. Stimulation of uncultured WB (AML and Healthy) with LAA /SEB increases frequencies of (antigen-specific) degranulating and intracellularly IFNγ–producing immune cells

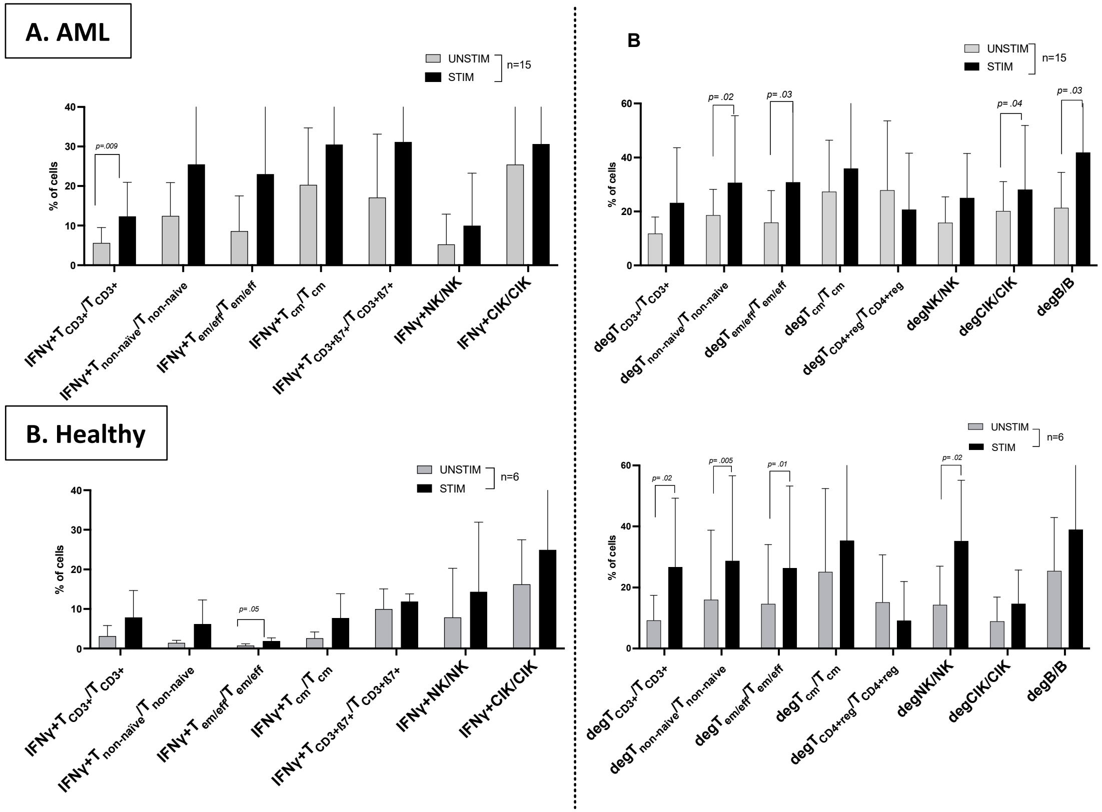

Mean frequencies ± standard deviation of uncultured immune cells producing intracellular IFNγ (Figure 1A) or expressing CD107a (Figure 1B) or in leukemic patients' WB (above) and healthy donors' WB (below) with LAA stimulation are provided; Data normality was assessed using the Shapiro–Wilk test. Statistical analyses were conducted using *t*-tests: Differences were considered as highly significant with p values ≤ 0.005, as significant with p values ≤0.05, and as borderline significant with p values between 0.05 to 0.10. Abbreviations of cell types are given in *Table 2*.

Figure S3. (Leukemia) derived DC/DC$_{leu}$ are increased in AML or healthy WB under the influence of Kit M.

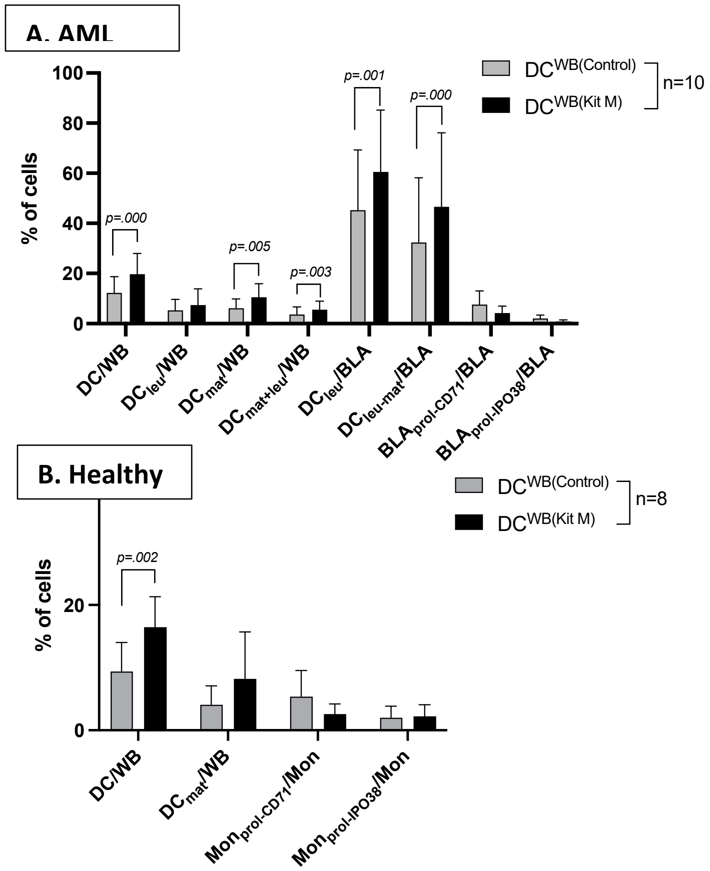

Mean frequencies ± standard deviation of generated DC subtypes and proliferating blasts in leukemic patients' WB and (Figure 2A) generated DC subtypes and monocytes in healthy donors' WB (Figure 2B) (with and without Kit M pretreated WB ($DC^{WB(Control)}$; $DC^{WB\ (Kit\ M)}$). Data normality was assessed using the Shapiro–Wilk test. Statistical analyses were conducted using *t*-tests: Differences were considered as highly significant with p values ≤ 0.005, as significant with p values ≤0.05, and as borderline significant with p values between 0.05 to 0.10. Abbreviations of cell types are given in *Table 2*.

Figure S4. Activated and memory T cells are increased after MLC with Kit M pretreated vs not pretreated patients' or healthy donors' WB

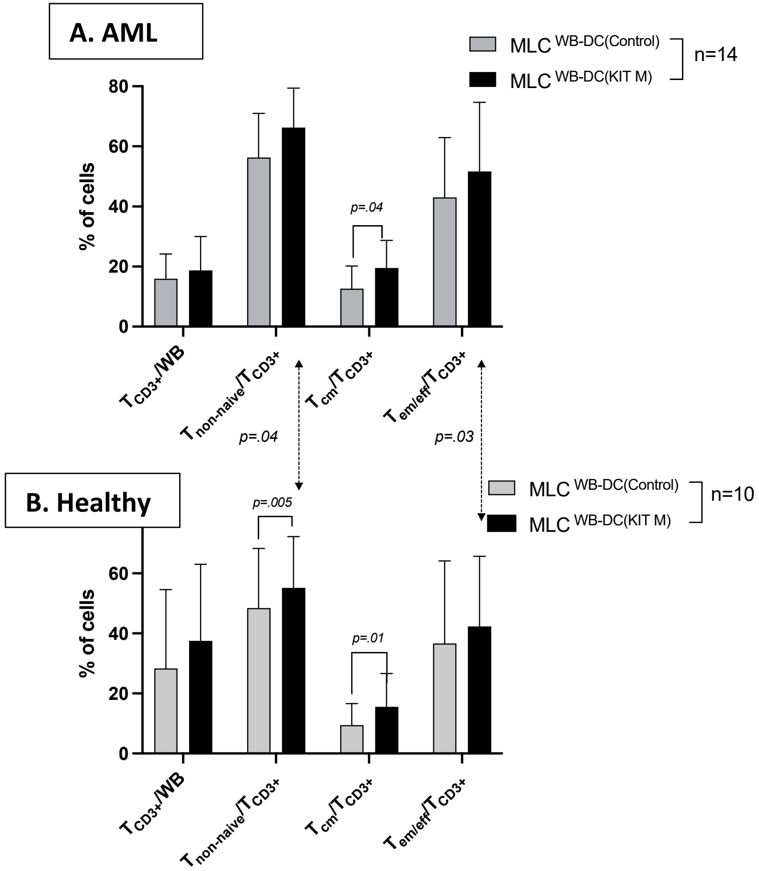

Mean frequencies ± standard deviation of T cell subsets after stimulation of T cell-enriched immunoreactive cells containing Kit M-pretreated WB (MLC $^{\text{WB-DC(Kit M)}}$) from leukemic (Figure 3A) or healthy (Figure 3B) T cells compared to WB not pretreated with Kit M (MLC $^{\text{WB-DC(Control)}}$). Data normality was assessed using the Shapiro–Wilk test. Statistical analyses were conducted using *t*-tests: Differences were considered as highly significant with p values ≤ 0.005, as significant with p values ≤0.05, and as borderline significant with p values between 0.05 to 0.10. Double-sided arrows give (significant) differences between defined cell subtypes in AML patients and Healthy donors. Abbreviations of cell types are given in *Table 2*.

Figure S5. Increased intracellular IFNγ-producing and degranulating immune cells after MLC in Kit-M-treated (*vs.* untreated) AML and healthy donors

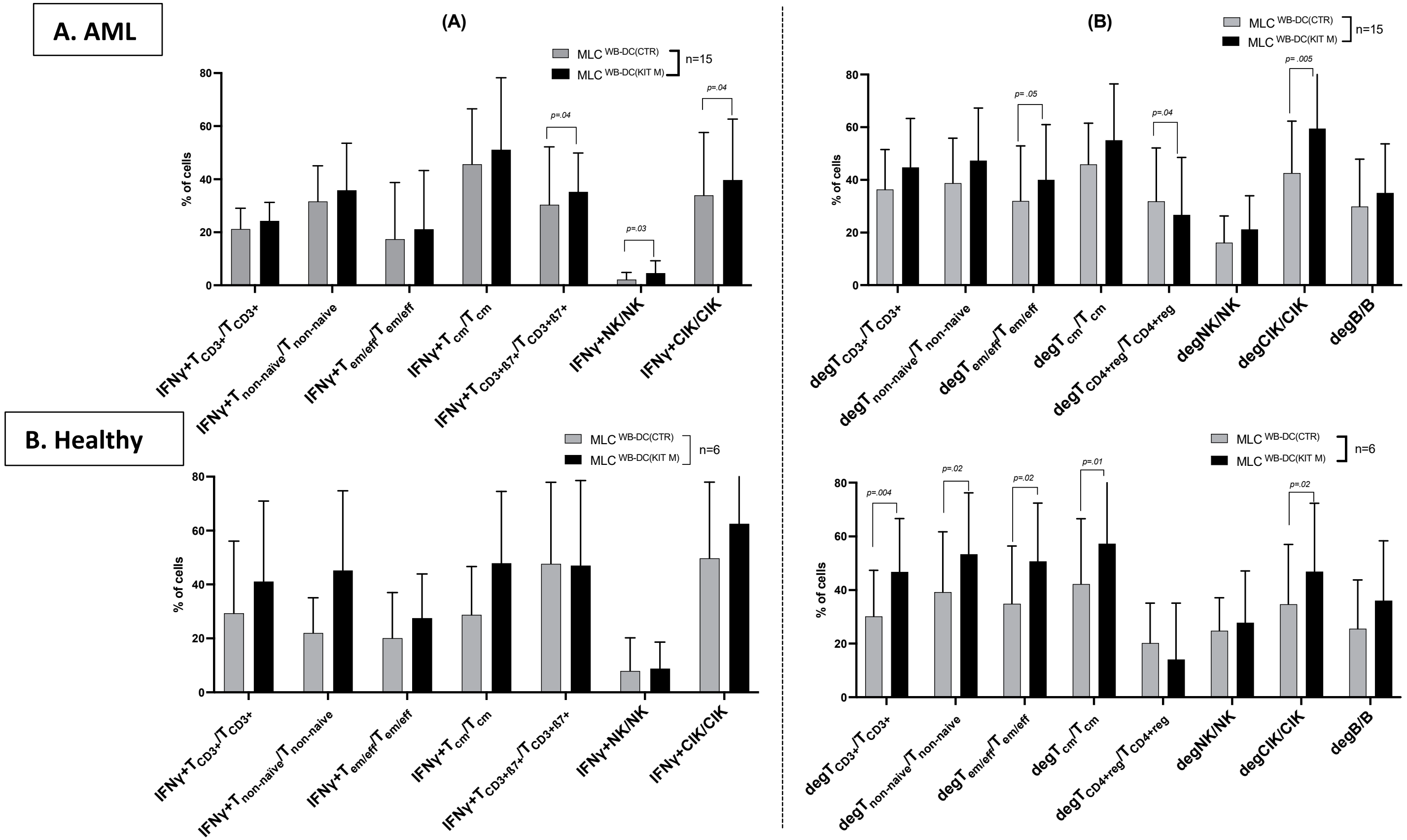

Mean frequencies ± standard deviation of Kit M-pretreated immune cells ($MLC^{WB\text{-}DC(CTR)}$, $MLC^{WB\text{-}DC(KIT\ M)}$) producing intracellular IFNγ (Figure 4A) or expressing CD107a (Figure 4B) in leukemic patients' WB (above) and healthy donors' WB (below) are provided; Data normality was assessed using the Shapiro–Wilk test. Statistical analyses were conducted using *t*-tests: Differences were considered as highly significant with *p* values ≤ 0.005, as significant with *p* values ≤0.05, and as borderline significant with *p* values between 0.05 to 0.10. Abbreviations of cell types are given in *Table 2*.

Figure S6. Correlations of ICM/ICML expressing cells with ex vivo functionality (A, B)

(A) Low frequencies of ICM/ICML expressing uncultured AML patients' T-cells correlate with ex vivo later-on achieved blast lysis and in vivo response to relapse therapy.

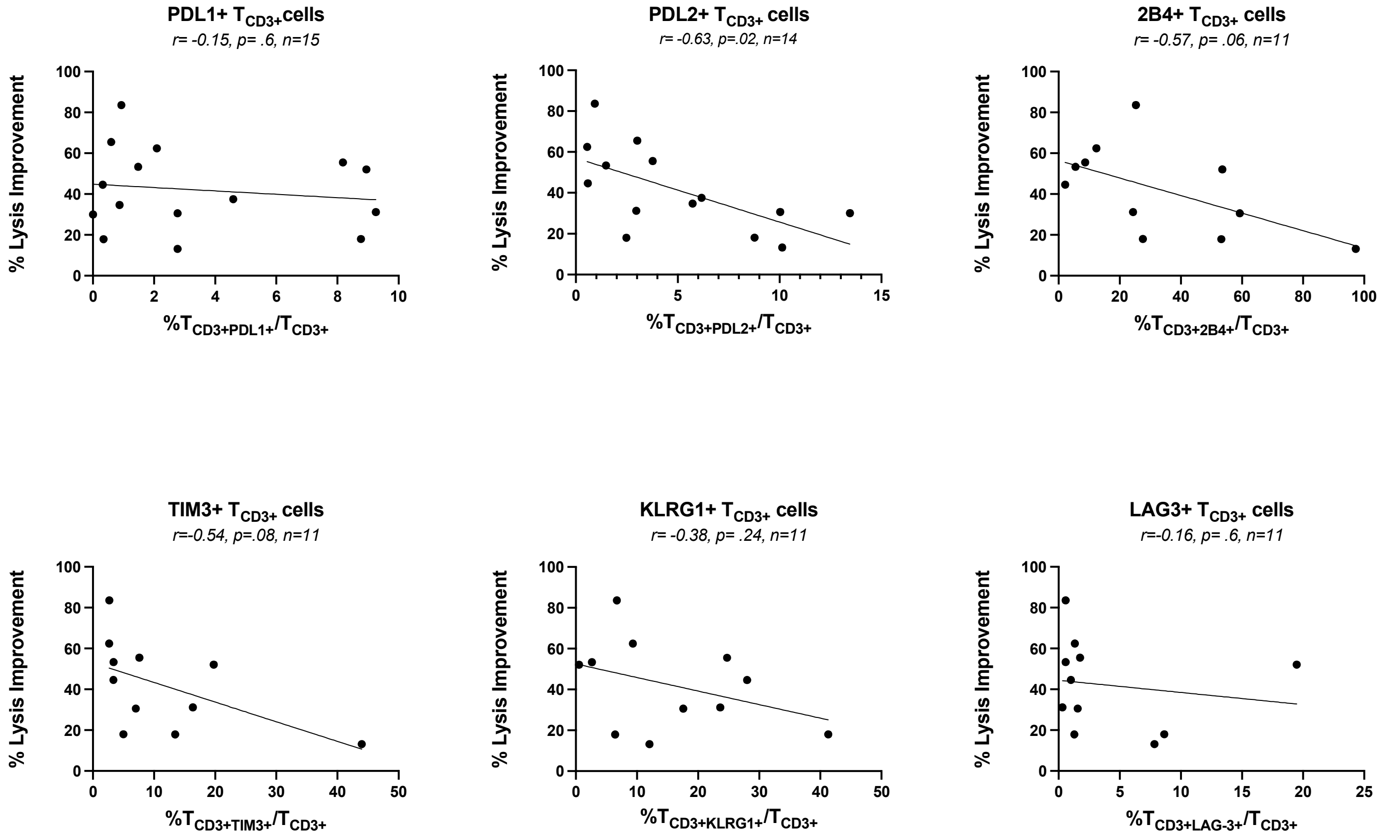

(B) Frequencies of ICM/ICML expressing cultured T cells after Kit M pretreated MLC did not correlate with ex vivo achieved blast lysis.

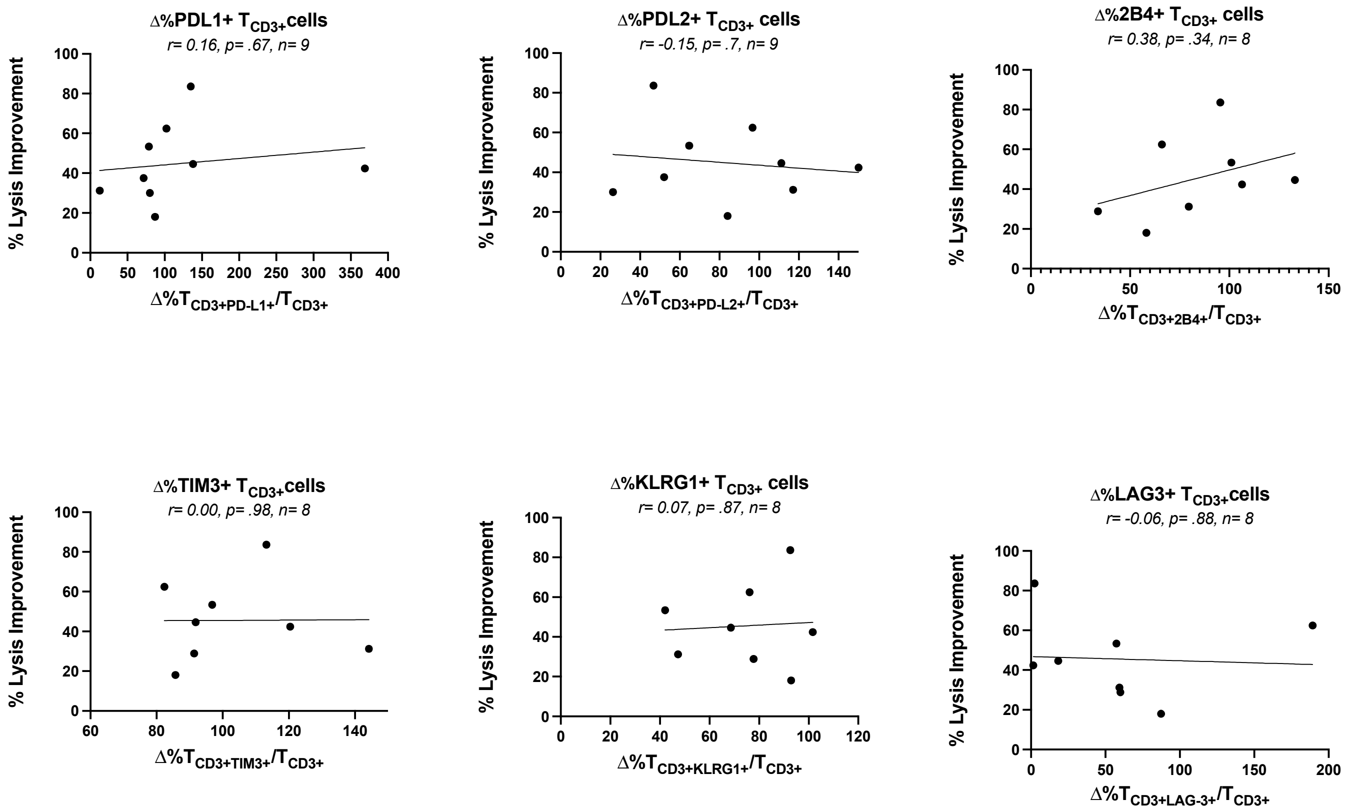

r- correlation coefficient, p- significance, n- number of cases. The average frequencies ± standard deviation of ICM/ICML expression on uncultured leukemic samples. Data normality was assessed using the Shapiro–Wilk test. Statistical analyses were conducted using *t*-tests: Differences were considered as highly significant with p values ≤ 0.005, as significant with p values ≤0.05, and as borderline significant with p values between 0.05 to 0.10. Abbreviations of cell types are given in *Table 2*.

Figure S7. Increased antileukemic cytotoxicity after MLC in Kit-M-treated (*vs.* untreated) AML patients' WB

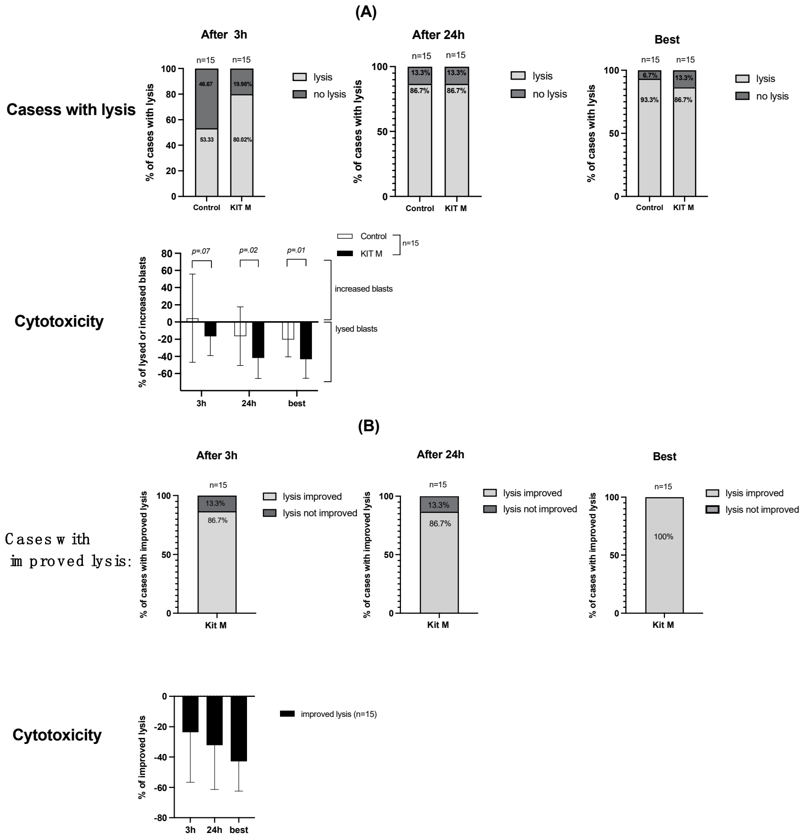

Stimulatory effect of Kit M-pretreated (vs. untreated) WB on the cytotoxic activity after 3 h and 24 h of co-culture of immunoreactivity cells ('effector cells') and blasts ('target cells'). Given are the proportions of cases with blast lysis and the frequencies ± standard deviation of increased or lysed blasts after $MLC^{WB\text{-}DC(Kit\ M)}$ (Kit M) compared to $MLC^{WB\text{-}DC(Control)}$ (Control) after 3h and 24h and the 'best' achieved blast lysis after 3h or 24h (Figure. 5A); Given are the proportions of cases with an improvement in blast lysis and the frequencies ± standard deviation of improved lysed blasts after $MLC^{WB\text{-}DC(Kit\ M)}$ in relation to $MLC^{WB\text{-}DC(Control)}$ after 3h and 24h and the 'best' achieved improvement in blast lysis after 3h or 24h (Figure. 5B). Data normality was assessed using the Shapiro–Wilk test. Statistical analyses were conducted using *t*-tests: Differences were considered as highly significant with p values ≤ 0.005, as significant with p values ≤0.05, and as borderline significant with p values between 0.05 to 0.10.

Materials and Methods

Patients´ characteristics

The cellular composition of blood samples from AML patients was 13.28% blasts (range 0.65–60.97), 11.46% T cells (range 3–19), 4.76% NK cells (range 1–8.67), and 1.07% CIK cells (range 0.38–3.68). The cellular composition of blood samples from healthy volunteers was 5.46 % monocytes (range 1.19–11.4), 18% T cells (range 15.5–20.11), 6.67% NK cells (range 2.85–7), and 6.5% CIK cells (range 0.98–8.99). In cases of aberrant expression of lineage markers on blasts, these markers were not included.

Flow Cytometry

Using a refined gating technique and the analysis software Cell Quest-Pro (Becton Dickinson, Heidelberg, Germany), the functionalities of cells (proliferation, cytokine production, degranulation, and cytotoxicity) could be investigated. Panels with various monoclonal antibodies (moAbs) labelled with fluorescein isothiocyanate (FITC), phycoerythrin (PE), phycoerythrin cyanin 7 (PCy7) or allophycocyanin (APC) were used, provided by Beckman Coulter[a] (Krefeld, Germany), Becton Dickinson[b] (Heidelberg, Germany), Bio Legend[c] (Amsterdam, Netherlands), Miltenyi Biotec[d] (Bergisch Gladbach, Germany), and Santa Cruz Biotechnology[e] (Heidelberg, Germany). Cells were stained with FITC-conjugated moAbs CD3[b], CD4[b], CD25[a], CD33[a], CD34 [a], CD71 [a], CD107 [c], CD117 [c], CD154 [b], CD45RO [a], IPO38 [e], CD274 (PDL1) [b], CD244 (2B4) [c]; PE-conjugated moAbs CD3 [d],CD4 [b], CD56 [a], CD80 [a], CD117 [a], CD127 [a], CD152(CTLA4) [b], CD279(PD1) [b], CD206 [a], 6B11 [b], IFNγ [c], TCRγδ [b], CD244 (2B4) [c], TIGIT [c], TIM3 [c]; PC7-conjugated moAbs CD3 [a], CD4 [a], CD25 [b], CD33 [a], CD34 [a], CD56 [a], CD117 [a], CD197 [b], TNFα [c], KLRG1 [c] and APC- conjugated moAbs CD3 [a], CD33 [a], CD34 [a], CD56 [a], CD80 [c], CD117 [a], CD137 [b], CD206 [b], CD45RO [c], Integrinβ7 [b], CD273(PDL2)[b], TIM3 [c],LAG3 [c]. Non-viable cells were detected using 7AAD [b]. Isotype controls were conducted according to the manufacturer's instructions.

Before staining, erythrocytes in WB samples were lysed with lysing buffer (Becton Dickinson). Cells were then incubated with the corresponding moAbs for 15 min in the dark using a staining medium containing 95% PBS and 5% FCS (Biochrom, Berlin, Germany). Intracellular staining (IPO38, IFNγ) was conducted with the FIX&PERMTM Cell Fixation and Permeabilization Kit (Thermo Fisher Scientific, Darmstadt, Germany). Evaluation and quantification of stained cells was obtained with the fluorescence-activated cell sorting flow cytometer FACS Calibur (Becton Dickinson) and the analysis software Cell Quest-Pro 6.1 (Becton Dickinson), applying a refined gating strategy [17].

Sample Preparation

Peripheral blood mononuclear cells (PBMC) were isolated from the WB using the Ficoll-Hypaque density gradient centrifugation (Biocoll separating solution, Biochrom, Berlin, Germany). Then, T cells were isolated from PBMCs via MACS microbead technology, based on a CD3 magnetic cell selection (CD3 Microbeads, Miltenyi Biotech, Bergisch Gladbach, Germany), as described in the manufacturer's instructions. PBMC and T cells were frozen with 70% RPMI-1640 (Biochrom) containing 100 U/mL penicillin and 0.1 mg/mL streptomycin (PAN-Biotech, Aidenbach, Germany) (RPMI/PS), 20% human serum (PAN-Biotech), and 10% dimethyl sulfoxide (Sigma Aldrich Chemie GmbH, Steinheim, Germany) and stored at −80 °C until use.

Dendritic Cell Culture (DCC)

WB cultures were incubated for 7–8 days under physiological conditions (37 °C, 5% CO_2, 21% O_2, and 95% humidity). Flow cytometric analyses of DC-subtypes and proliferating blasts from both Kit-M-treated WB ($DC^{WB(Kit M)}$) and untreated WB ($DC^{WB(Control)}$) were performed before and after culture using a refined gating strategy. [17].

T Cell-Enriched Mixed Lymphocyte Culture (MLC)

To generate T cell-enriched immune-reactive cells, thawed autologous T cells were stimulated with generated DC/DC_{leu}. Specifically, $1x10^6$ T cells and a fraction of DCC containing $2.5x10^5$ DC/DC_{leu} were co-cultured in 24-multiwell-tissue-culture-plates, with the total volume of the cell cultures being adjusted to 1ml with RPMI-1640-medium (Biochrom) containing 100U/ml penicillin (Biochrom). 50U/ml IL-2 (Peprotech) were added to all cultures ($MLC^{WB-DC(Kit M, Control)}$) on day 0 and day 2-3. Under physiological conditions (37 °C, 5% CO_2, 21% O_2, and 95% humidity), the MLC was incubated for 7 days. After culture, measurements were carried out with Kit M ($MLC^{WB-DC(Kit M)}$) and Control ($MLC^{WB-DC(Control)}$) [17].

Intracellular Cytokine Assay (INTCYT)

The cells were stained with PE-conjugated IFNγ antibodies (BioLegend, San Diego, CA, USA). In analogy to the DEG, only uncultured AML samples were stimulated with LAA. To avoid spontaneous cytokine secretion, 5 μg/mL Brefeldin A solution (Bio Legend) was added. The cultures were incubated for 16 h at 37 °C, 21% O_2, and 10% CO_2. After harvest, centrifuged, resuspended in PBS/FCS, and stained with antibodies, and then analyzed by flow cytometry. [18].

Degranulation Assay (DEG)

AML samples were stimulated with two leukaemia-associated antigens (LAA): 2 µg/mL "Wilms Tumor 1" (PepTivator®WT1, Miltenyi Biotec) and 2 µg/mL "Preferentially Expressed Antigen of Melanoma" (PepTivator®PRAME, Miltenyi Biotec). Healthy samples were stimulated/not stimulated with 10 µg/mL staphylococcal enterotoxin B (SEB, Sigma-Aldrich, St. Louis, MO, USA). Cultures without antigen stimulation served as a negative Control ('Unstimulated'). After an incubation of 16 h at 37 °C, 21% O_2, and 10% CO_2, cells were harvested, stained, and analyzed by flow cytometry [18].

Cytotoxicity Fluorolysis Assay (CTX)

A fraction of $MLC^{WB-DC\ (Kit\ M)\ (Control)}$ containing $1x10^6$ T cells (effector cells) was co-cultured with $1x10^6$ thawed autologous leukaemic blasts (target cells) for 3 and 24h at 37°C, 21% O_2, 5% CO_2. Target cells were stained with respective antibodies before incubation. After harvest, 7AAD and a defined number of Fluorosphere beads (Beckman Coulter) were added. The lytic activity of effector cells was calculated and defined as the percentage of viable target cells in the culture with co-cultured effector and target cells (for 3h and 24h), as compared to the Control. As a control, effector and target cells were cultured separately and mixed shortly before measurements. Flow cytometric analyses were performed using a refined gating strategy. Achieved blast lytic activity was defined as the percentage difference of viable target cells (blasts) between the effector-target-cell culture and the control. [17].

17. Klauer, L.K.; Schutti, O.; Ugur, S.; Doraneh-Gard, F.; Amberger, D.C.; Rogers, N.; Krämer, D.; Rank, A.; Schmid, C.; Eiz-Vesper, B.; et al. Interferon Gamma Secretion of Adaptive and Innate Immune Cells as a Parameter to Describe Leukaemia-Derived Dendritic-Cell-Mediated Immune Responses in Acute Myeloid Leukaemia in vitro. *Transfus. Med. Hemother.* **2022**, *49*, 44–61, doi:10.1159/000516886.
18. Schutti, O.; Klauer, L.; Baudrexler, T.; Burkert, F.; Schmohl, J.; Hentrich, M.; Bojko, P.; Kraemer, D.; Rank, A.; Schmid, C.; et al. Effective and Successful Quantification of Leukemia-Specific Immune Cells in AML Patients' Blood or Culture, Focusing on Intracellular Cytokine and Degranulation Assays. *Int. J. Mol. Sci.* **2024**, *25*, doi:10.3390/ijms25136983.
